# Successful Implementation of Extracorporeal Membrane Oxygenation Support as a Bridge to Heart-Lung Transplantation in an Eisenmenger’s Syndrome Patient With Paradoxical Coronary Embolism

**DOI:** 10.1177/2324709619846575

**Published:** 2019-05-03

**Authors:** James Zhang, Sumit Patel, Leonardo Clavijo, David Laughrun

**Affiliations:** 1 University of Southern California, Los Angeles, CA, USA

**Keywords:** Eisenmenger’s syndrome, ECMO, heart-lung transplantation

## Abstract

We report a case of a 23-year-old female with a history of unrepaired ventricular septal defect and pulmonary arterial hypertension with Eisenmenger’s syndrome (ES) presenting with chest pain. Electrocardiography demonstrated new anterior Q waves and anterolateral ST elevations, and coronary angiography revealed a large organized thrombus in the mid-left anterior descending artery consistent with paradoxical coronary embolism. Patient was treated with percutaneous coronary intervention and aggressive anticoagulation management. Intensive care unit course was complicated by respiratory failure requiring intubation due to hospital-acquired pneumonia in the setting of severe pulmonary hypertension. Patient was emergently initiated on veno-venous extracorporeal membrane oxygenation support (ECMO) as a bridge to heart-lung transplantation. After initiation of ECMO, patient displayed significant clinical improvement and underwent successful heart-lung transplantation. This case highlights veno-venous ECMO as a bridge to heart-lung transplantation in acutely decompensated patients with ES, and is the first reported case of paradoxical coronary embolism in a patient with ES.

## Introduction

Eisenmenger’s syndrome (ES) may develop as a sequelae of congenital heart disease and is defined by the presence of cyanosis due to right-to-left shunting in the setting of severe pulmonary hypertension. In patients with advanced disease or severe symptoms, evaluation for heart-lung transplantation, or heart repair with lung transplantation, should be explored. Indications for mechanical circulatory support such as extracorporeal membrane oxygenation support (ECMO) in ES patients are not well defined. This case suggests that veno-venous (VV) ECMO may be a viable option as a bridge to heart-lung transplantation in acutely decompensated patients.

## Case Presentation

We report a case of a 23-year-old female with a history of unrepaired ventricular septal defect (VSD) and pulmonary arterial hypertension with ES presenting with chest pain and shortness of breath. She was diagnosed with a membranous VSD at the age of 3 years and was noted to have pulmonary hypertension at the age of 8 years. She had self-discontinued all medications at 18 years of age. She had no history of prior thrombotic events and was not taking oral contraceptives.

**Figure 1.**
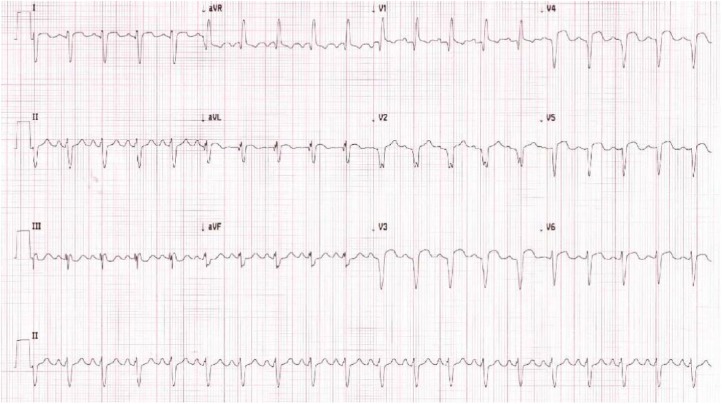
Admission electrocardiography with anterior Q waves and anterolateral ST elevations, consistent with myocardial infarction.

Patient presented with sudden-onset, left-sided, pleuritic chest pressure radiating to the back with shortness of breath at rest. She had baseline 1 pillow orthopnea, but denied lower extremity edema and paroxysmal nocturnal dyspnea. On admission, she was found to be in moderate respiratory distress on high-flow nasal cannula with a 2/6 holosystolic murmur on cardiac auscultation. Blood pressure was 93/54 mm Hg, heart rate was 105 beats/min, and oxygen saturation was 96% on 70% high-flow oxygen. Laboratory findings revealed an elevated troponin greater than 50 ng/mL, brain natriuretic peptide of 7,575 pg/mL, and hematocrit of 38.6%. Electrocardiography on admission demonstrated new anterior Q waves and anterolateral ST elevations (Figure 1). Bedside echocardiography revealed moderate hypokinesis of the basal to mid anterolateral wall. Patient was admitted to the medical intensive care unit.

**Figure 2.**
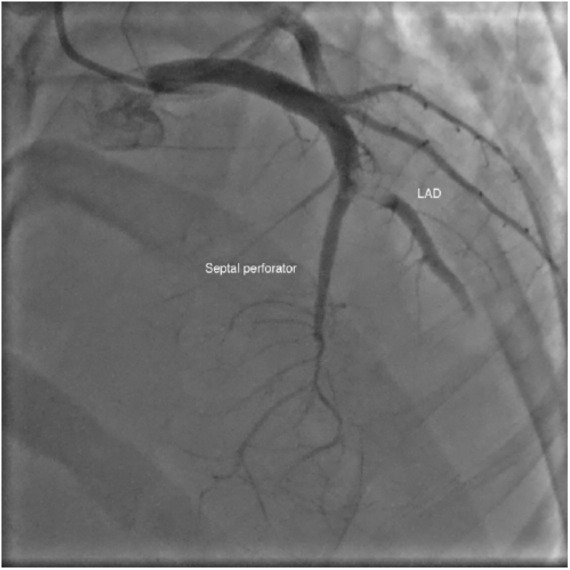
Coronary angiography demonstrating a large, organized thrombus in the mid-left anterior descending (LAD) artery near the junction of a large septal perforating artery.

**Figure 3.**
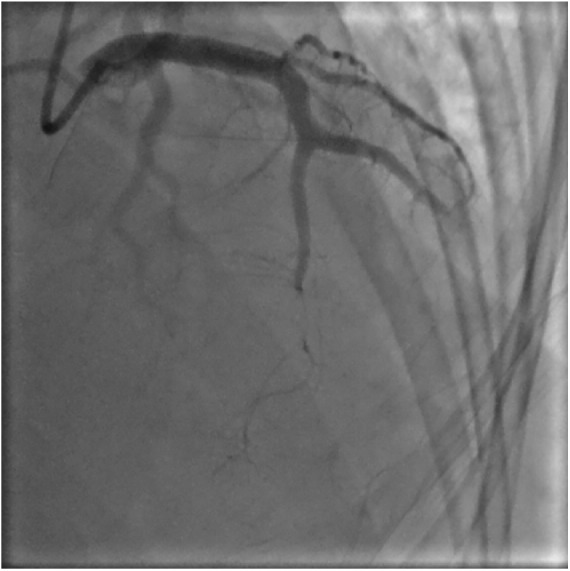
Coronary angiography following aspiration and mechanical thrombectomy. Distal embolization of thrombus in the left anterior descending and septal perforating artery is seen.

Coronary angiography revealed a large organized thrombus in the mid-left anterior descending artery near the junction of a large septal perforator (Figure 2). Patient underwent manual aspiration and thrombectomy with balloon angioplasty. There was distal embolization of thrombus in both the left anterior descending and a large septal perforator, seen on later angiography images (Figure 3). There was no apparent atherosclerotic coronary artery disease. Right heart catheterization revealed central venous pressure 14 mm Hg, pulmonary artery pressure 105/58 mm Hg (mean pulmonary arterial pressure 72 mm Hg), and pulmonary capillary wedge pressure 35 mm Hg. Computed tomography pulmonary angiography demonstrated a dilated main pulmonary artery of 4 cm with no evidence of pulmonary embolism. Ultrasound Doppler of the extremities showed no evidence of deep vein thrombosis. Post-intervention echocardiography revealed an ejection fraction of 45%. Patient was placed on intravenous eptifibatide and continuous heparin infusion.

During intensive care unit course, the patient developed worsening respiratory compromise with desaturations on high-flow nasal cannula and bilevel positive airway pressure. Chest X-ray revealed new bilateral consolidations with concern for hospital-acquired pneumonia and impending acute respiratory distress syndrome. Patient was initiated on inhaled nitrous oxide, intravenous epoprostenol, and continued on macitentan and sildenafil. She required vasopressor therapy for hemodynamic support. Despite up-titration of pulmonary vasodilatory medications, she remained hypoxemic on bilevel positive airway pressure. She was intubated and emergently placed on VV ECMO support at a flow rate of 7 L/min. Hemodynamic parameters and oxygenation improved. Nitrous oxide was successfully weaned with down-titration of vasopressor therapy. Patient was accepted and transferred via emergent air transport to an outside hospital where she underwent successful heart-lung transplantation. Patient was seen in outpatient follow-up clinic at our institution and was ambulatory, independently living, and feeling well.

## Discussion

Paradoxical embolism refers to a venous thromboembolism that passes into the systemic circulation via an intracardiac defect or shunt. The risk of paradoxical embolism is increased in conditions that cause elevation of right-sided cardiac pressures with right-to-left shunting, such as ES.^1^ Paradoxical coronary embolism is rare and accounts for only 10% to 15% of paradoxical emboli events.^2^ It should be suspected in young individuals at low risk for atherosclerotic disease and without other risk factors for coronary thrombosis such as oral contraceptive use and polycythemia. The management of paradoxical coronary embolism is highlighted in this case, which includes aspiration thrombectomy with or without angioplasty and aggressive anticoagulation management. To our knowledge this is the first reported case of paradoxical coronary embolism in a patient with ES.

The use of mechanical circulatory support such as ECMO in ES is not well studied. In this particular case, refractory hypoxia despite maximum vasodilatory therapy in the setting of pulmonary hypertension and hospital-acquired pneumonia led to the initiation of ECMO. While there have been previous case reports of successful utilization of ECMO in patients with ES, this case highlights the first known implementation of ECMO as a bridge to heart-lung transplantation.

The decision to utilize ECMO in ES patients poses unique challenges that should be thoroughly considered prior to initiation.^3^ Patients with ES have baseline thrombocytopenia and coagulopathy, increasing the risk of hemorrhagic events, along with other hematologic consequences of ECMO including hemolysis, thromboembolic events, and disseminated intravascular coagulation. The increased risk of infection should also be considered, as it may lead to sepsis and further hemodynamic compromise.

The 2 basic support modes for ECMO include veno-arterial (VA) for both respiratory and hemodynamic support, and VV for primarily respiratory support. VV ECMO carries less bleeding risk with a 17% rate of hemorrhage versus 34% in VA ECMO.^4^ The optimal mode of support in ES patients has not been established. While severe pulmonary hypertension and impending right ventricular failure is typically an indication to initiate VA ECMO, VV ECMO has been utilized successfully in patients with a patent foramen ovale or atrial septal defect.^5,6^ It has been postulated that a sufficient fraction of mechanically oxygenated blood is shunted through the defect, leading to improvement in systemic oxygenation and left ventricular filling.^7^ This is supported by our case, as initiation of VV ECMO in a VSD patient led to rapid improvement in systemic oxygenation and hemodynamics, with eventual down-titration of vasodilatory support. Furthermore, initiation of VV ECMO may contribute to right ventricular offloading,^8^ demonstrated in our case by a decrease in mean pulmonary arterial pressure from 76 to 67 less than 24 hours after initiation of ECMO.

## Conclusion

The optimal timing and indications for ECMO support in Eisenmenger syndrome are not well defined. Prompt initiation of VV ECMO following respiratory compromise was vital to a successful bridge to heart-lung transplant strategy in our patient.
